# Enhanced ionic conductivity and mechanical strength in nanocomposite electrolytes with nonlinear polymer architectures

**DOI:** 10.55730/1300-0527.3533

**Published:** 2022-12-12

**Authors:** Recep BAKAR, Saeid DARVISHI, Erkan ŞENSES

**Affiliations:** 1 Department of Material Science and Engineering, Koç University, İstanbul, Turkey; 2 Department of Chemical and Biological Engineering, Koç University, İstanbul, Turkey; 3 Boron and Advanced Materials Application and Research Center (KUBAM), İstanbul, Turkey; 4 Koç University Surface Science and Technology Center (KUYTAM), İstanbul, Turkey

**Keywords:** Polymer electrolytes, poly(ethylene oxide), silica nanoparticles, polymer architecture, ionic conductivity, Arrhenius behavior

## Abstract

Solvent-free polymer-based electrolytes (SPEs) have gained significant attention to realize safer and flexible lithium-ion batteries. Among all polymers used for preparing SPEs electrolytes, poly(ethylene oxide), a biocompatible and biodegradable polymer, has been the most prevalent one mainly because of its high ionic conductivity in the molten state, the capability for the dissolution of a wide range of different lithium salts as well as its potential for the environmental health and safety. However, linear PEO is highly semicrystalline at room temperature and thus exhibits weak mechanical performance. Addition of nanoparticles enhances the mechanical strength and effectively decreases the crystallization of linear PEO, yet enhancement in mechanical performance often results in decreased ionic conductivity when compared to the neat linear PEO-based electrolytes; new strategies for decoupling ionic conductivity from mechanical reinforcement are urgently needed. Herein, we used lithium bis(trifluoromethane-sulfonyl)-imide (LiTFSI) salts dissolved in various nonlinear PEO architectures, including stars (4-arms and 8-arms) and hyperbranched matrices, and SiO_2_ nanoparticles (approximately equal to 50 nm diameter) as fillers. Compared to the linear PEO chains, the room temperature crystallinity was eliminated in the branched PEO architectures. The electrolytes with good dispersion of the nanoparticles in the nonlinear PEOs significantly enhanced ionic conductivity, specifically by approximately equal to 40% for 8-arm star, approximately equal to 28% for 4-arms star, and approximately equal to %16 for hyperbranched matrices, with respect to the composite electrolyte with the linear matrix. Additionally, the rheological results of the SPEs with branched architectures show more than three orders of magnitude enhancement in the low-frequency moduli compared to the neat linear PEO/Li systems. The obtained results demonstrate that the solvent-free composite electrolytes made of branched PEO architectures can be quite promising especially for irregularly shaped and environmentally benign battery applications suitable for medical implants, wearable devices, and stretchable electronics, which require biodegradability and biocompatibility.

## 1. Introduction

The development of better, safer, and sustainable power sources has been one of the most important technological challenges because of ever-growing increase in requirement for packed, lightweight, and mobile consumer electronics and energy storage devices [1–3]. To satisfy the demand, chemical energy storage in batteries appears to be the most promising and widely used method when compared to conventional technologies such as pumped hydroelectric or compressed air, and flywheel [1–3]. Batteries, with their high energy density with a variety of current and voltage ranges, can be utilized for both stationary and mobile applications due to their packed and lightweight structure [4, 5]. Despite environmental and sustainability risks related to toxic and rare cathode materials (lithium, cobalt, or nickel), volatility of the organic solvent-based electrolytes, and greater CO_2_ emissions due to heavier specific weights of the liquid electrolytes than water-based electrolytes [4, 5] when compared to zinc–carbon and zinc/alkaline manganese dioxide based primary batteries, [6–9] lithium-ion batteries (LIBs) have increasingly attracted more attention due to their low maintenance, high energy density, prolonged cycling stability, longer shelf life, electrochemical potential, and their recyclability [4, 10–13].

The commercial LIBs mostly use liquid electrolytes with high ionic conductivities [11]; however, they are often highly volatile, flammable, and toxic, which may result in environmental and safety hazards such as fire, leakage, explosion, short circuit, and lithium dendrite formation, which endangers battery safety and human and environmental health [10, 11, 14, 15]. To overcome these disadvantages, solid polymer electrolytes (SPEs) have gained significant attention [10, 11, 15, 16]. SPEs, in general, provide many advantages over liquid electrolytes, including higher flexibility, greater energy density and processability, good thermo-mechanical stability, nonflammability, and wide electrochemical stability, leading to more compact and safer batteries [4, 10, 13, 16–18]. However, their commercial use is limited due to their low ionic conductivity, e.g., room temperature ionic conductivities of SPEs are (10^−5^ S/cm) at least 2–3 orders of magnitude lower than common liquid electrolytes (approximately 10^−2^ S/cm) [13].

Among the water-soluble polymers including polyacrylamides, polyacrylic acid, and polyvinyl alcohol, poly(ethylene oxide) (PEO)-based SPEs have been a common choice due to various reasons. PEO has very low glass transition temperature (T_g_ approximately 220 K) when compared to other water-soluble polymers such as polyacrylamides (T_g_ approximately 450 K) [19], polyacrylic acid (T_g_ approximately 390 K) [20], and polyvinyl alcohol (T_g_ approximately 350 K) [21], which allows fast segmental relaxation (and hence ion transport) [13] and easy processability at room temperature [22–24]. Moreover, as opposed to polyacrylamides, PEO is a biodegradable polymer, an important feature for battery implants and wearable electronics [23–25] and provides higher thermal decomposition temperature compared to polyacrylic acid and polyvinyl alcohol [20, 25–27]. From the practical point of view, PEO can solve a variety of different salts through the complexation between ether-oxygen and the lithium ions [13, 28, 29], and its electrolytes properties in the neat form have been well studied, thus allowing for a meaningful evaluation of conductivity performance of the electrolytes.

As ion transport primarily occurs in the amorphous phase [13, 30–32], inherently high degree of crystallinity of PEO impedes ionic mobility at room temperature, resulting in low conductivities (approximately 10^−6^ to 10^−8^ S/cm) [13]. Above its melting temperature (T_m_ = 333 K), the conductivity of the molten PEO electrolyte is increased up to the values ranging around 10^−3^ S/cm, yet the decrease in the mechanical stability limits its use as a solid-state electrolyte [33]. Enhancing ionic conductivity of PEO electrolytes without much sacrificing, or even improving, the mechanical strength remains as a major challenge. In this regard, incorporation of nanoparticles into the electrolytes to form composite electrolytes is a promising approach [30, 34, 35]. For example, Utpalla et al. [34] studied the addition of silica nanoparticles into PEO and reported that enhanced free volume using inorganic nanofillers improved the ionic conductivity in PEO/silica-based copolymer electrolytes. Similarly, Michael et al. [30] revealed incorporation of nanofillers into PEO matrix increased free volume by decreasing crystallization and hence facilitated ion migration. Schaefer and coworkers [35] incorporated the SiO_2_ nanoparticles (NPs) grafted by the PEO in the composite electrolyte, which resulted in drastically higher mechanical modulus along with high conductivity because of SiO_2_ core fractions and increasing free volume, respectively. All these studies on nanocomposite electrolytes aimed for enhancing ion mobility by impeding crystallization by adding NPs into linear polymer matrices; however, in most of the cases, the NP dispersion in salt containing matrices remained poor and was not controlled.

Different from the previous approaches, this study focuses on the polymer component by systematically increasing the degree of branching of the polymer chains and explores the possibility of using composites electrolytes with nonlinear matrices and well dispersed silica nanoparticles to achieve simultaneous improvement in the ionic conductivity and mechanical moduli of SPEs. This is rationalized by the fact that the free volume is enhanced by branching in the nonlinear topologies due to excessive number of chain ends compared to the linear chains [36, 37]. For example, Chremos and coworkers [37] studied star polymers of different number of arms and arm molar mass, and reported that increasing the arm number promoted the free volume and mobility compared the linear chains. Moreover, a variety of studies on the ‘neat’ PEO architectures including linear, stars, and hyperbranched also investigated the effect of polymer topology on crystallization [13, 38–41]. In this sense, Zardalidis et al. [38] studied the dependence of crystallization on topological changes using linear and ring PEO architectures and reported that the ring PEO possessed lower degree of crystallinity compared to the linear analogue due to its more restrained structure. In a similar work, Chen et al. [42] investigated crystallization behavior in linear and star PEO topologies (3-arms and 4-arms) and found the crystallinity of star polymers was significantly reduced. Similarly, the study conducted by Coppola and coworkers [39] using different PEO topologies (linear and stars) uncovered crystallization kinetics of the star topologies was slower than their linear analogues mainly because of high degree of branching with slowed diffusivity in stars. Furthermore, in different studies, Yao et al. [41] and Lee et al. [40] unveiled that the crystallization of hyperbranched poly(ethylene oxide) was effectively hindered compared to crystallization of the linear architecture. Very recently, a previous work of the authors [13] on the PEO/PMMA-based electrolytes using various architectures (linear, stars, hyperbranched, and bottle brush) showed that the degree of neat PEO crystallinity for different architectures severely decreased with the increasing number of branching. These previous works show that the degree of PEO crystallization could be decreased more effectively when nonlinear PEO architectures are applied, resulting in an increase in the amorphous fraction of the PEO; thus, the electrolytes prepared using branched PEO topologies could have higher ionic conduction.

**Figure 1:**
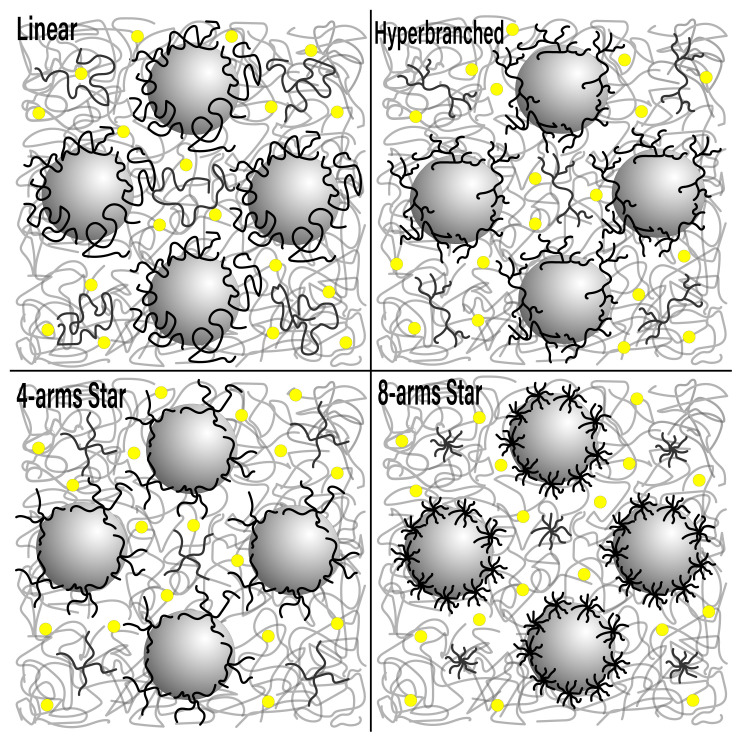
Schematic representation of the PNC based solid polymer electrolytes composing of PEO and Silica nanoparticles (big grey circles) with the architectural variations including linear, 4-arms star, 8-arms star, hyperbranched (3rd generation) used in this study (Yellow filled circle represents the Li^+^ ion).

In this study, the advantages provided by the enhanced free volume due to the increased number of end groups and suppressed crystallization arising from branching in the nonlinear PEO architectures are combined with the mechanical reinforcement due to the nanoparticles to obtain a synergistic effect on improved ionic conductivity and mechanical moduli. PEO with linear, 4-arms, 8-arms, and hyperbranched (3rd generation, 3G) architectures were employed to explore the role of polymer compactness on nanoparticle dispersion, ionic conductivity, and rheological behavior in polymer nanocomposite-based electrolytes, which was as schematically shown in Figure 1.

## 2. Materials and methods

Linear PEO, hyperbranched (3rd generation) PEO, and lithium bis(trifluoromethane)sulfonamide, LiTFSI, salt were purchased from Sigma-Aldrich. 4-arms star and 8-arms star PEO were supplied by Creative PEGWorks. All polymers were used as received without modification. Chemical structures of the polymers are provided in Figure S1. Table 1 displays their functionality, molar masses, and dispersities. The methyl ethyl ketone (MEK)-based colloidal spherical silica (diameter of 50 nm) solution was supplied by Nissan Chemical America Incorporation (USA).

### 2.1. Sample preparation

First PEO polymers with linear (MW approximately 20 kDa), 4-arms star (MW approximately 20 kDa), 8-arms star (MW approximately 20 kDa), and HB3G (MW approximately 20 kDa) architectures were dissolved in acetonitrile at 30 mg/mL concentration. The solutions were then stirred with a magnetic stirrer at room temperature for 6 h. Subsequently, the desired amount (to get the final 30 vol% NP concentration) of the colloidal silica solution was added into the polymer solutions, which was followed by another 6-h continuous stirring with a magnetic stirrer at room temperature. Next, a proper amount of lithium bis(trifluoromethane)sulfonamide, LiTFSI, was dissolved in acetonitrile in a glove box filled with Argon and the salt solution was stirred with a magnetic stirrer at room temperature for 6 h. Later, to prepare the PNC-based electrolytes, the desired amount of salt solution was added into the PEO/silica solution in the glovebox filled with Argon and then was stirred with a magnetic stirrer at room temperature for another 6 h. The solutions were then cast onto glass petri dishes and dried for approximately equal to 12 h at room temperature for overnight in a glove box filled with Argon. Finally, the dried films were annealed at 90 °C in a vacuum oven for 48 h to remove the residual solvents.

### 2.2. Characterization

#### 2.2.1. X-ray diffraction (XRD)

The XRD patterns of the pure PEOs and the electrolytes were recorded using the Bruker D8 Phaser – X-ray diffractometer with Cu Kα source at room temperature with the Bragg’s angles (2θ) varying from 5 to 60 degrees.

#### 2.2.2. Differential scanning calorimetry (DSC)

The differential scanning calorimetry (DSC) samples were prepared by putting approximately 8–10 mg of material in aluminum pans provided by TA Instruments. DSC experiments of the neat PEOs and its electrolytes were carried out with a TA Instruments DSC25 instrument equipped with a refrigerated cooling system. An aluminum pan was used as a reference. To get rid of the temperature history completely, all the samples were heated to 120 °C and waited there for 5 min. The samples were then quickly cooled down to −85 °C at the rate of 20 °C/min. DSC scans were then collected during the heating process to 120 °C at the rate of 10 °C/min.

#### 2.2.3. Electrochemical impedance spectroscopy (EIS)

Impedance characterization was carried out using an Autolab Potentiostat Galvanostat PGSTAT (Metrohm, Netherlands) in two-electrode configuration for PEO-based electrolytes. This arrangement was used to investigate electrode properties in solid-state systems. The measurement frequency was varied between 1 Hz to 1 MHz. Each SPE blend disc was sandwiched between two stainless steel blocking electrodes under argon environment in a glove box located at Koç University Boron and Advanced Materials Application and Research Center (KUBAM) and sealed in MTI Split Cell to measure the complex impedance spectra. After waiting for the temperature stabilization of 0.1 °C, the experiments were also carried out at various temperatures. The data was analyzed to characterize real and imaginary impedances using the NOVA software.

#### 2.2.4. SAXS measurements

The small-angle X-ray scattering (SAXS) data on molten nanocomposites were collected using Anton Paar’s SAXS Point 5.0 at Koç University. The samples were sealed between Kapton films and equilibrated at 80 °C (well above their melting temperatures) for 15 min before the measurements. The static SAXS profiles were used to evaluate the state of nanoparticle dispersion in the nanocomposites. Frames were collected for 5-min periods over a total duration of 15 min per sample. The transmission data was obtained for each sample and the Kapton background for proper reduction and subtraction. Data reduction was performed using the SAXS/WAXS analysis program of SAXSPoint 5.0.

#### 2.2.5. Rheology measurements

All rheological measurements in the melt state are performed using an Anton Paar MCR 302 rheometer equipped with a parallel geometry with diameter of 25 mm. For small-amplitude oscillatory shear (SAOS) measurements at 80 °C, first amplitude sweep test performed from 0.01% to 10% strain to find the linear viscoelastic region (LVR). The frequency sweep measurements were then performed between 0.01 and 100 rad/s at LVR.

## 3. Results and discussion

**Figure 2:**
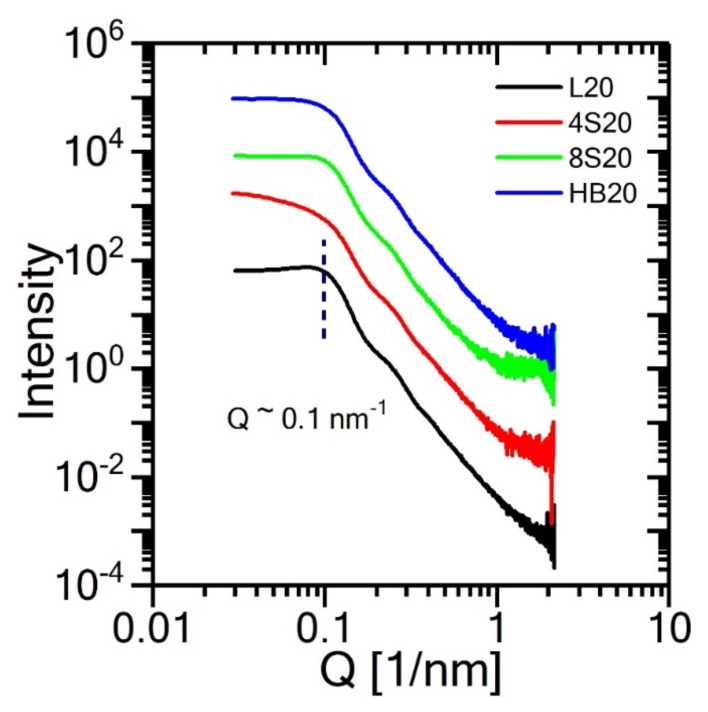
SAXS intensity profiles for the PEO-based PNCs electrolytes containing 30 vol% silica nanoparticles and LiTFSI (Li/EO=0.085 MR) with various PEO topologies. Profiles are shifted vertically for clarity.

NP agglomeration could lead nonhomogenous dispersion of the particles and adversely affect the characteristics of the composite electrolytes such as crystallization and ionic conductivity [34, 43–46]. The dispersion of silica nanoparticles in the PEO matrix was investigated by performing SAXS measurements in the molten state of the composite electrolytes. The intensity profiles as a function of scattering vector, *Q* = 4π sin θ/λ, (where 2θ is the angle between the incident and the scattered beam and λ is the wavelength of the X-rays) are shown in Figure 2 for all samples. At low *Q*, the intensity from all samples reaches *Q*-independent plateau, confirming the absence of large-scale agglomerates, hence individual NP dispersion in all samples. In addition, for the samples with linear, 8-arms star, and hyperbranched architectures, we observe the structure factor peaks at *Q* approximately equal to 0.1 nm^−1^ corresponding to the average Bragg spacing of 2π/*Q* approximately equal to 63 nm. This value is consistent with the theoretical prediction for the center-to-center interparticle distance (*h = d* [2/(πφ)]^1/3^ approximately equal to 64 nm) of spherical NPs of diameter *d* = 50 nm and 30 vol. % loading. This further supports the individual NP dispersion in each matrix. The peak intensity in nanocomposite samples with 4-arms matrix is not as sharp as in other samples possibly due to more uneven spacing between the dispersed NPs although the particles disperse individually (as evidenced by the lack of low-*Q* upturn in intensity). Overall, the NPs remain well-dispersed in all electrolyte samples; thus, the observed differences in the rheological behavior and ionic conduction of the composite electrolytes are not caused by the NP structuring, rather the difference in the architecture of the matrix polymers.

**Figure 3:**
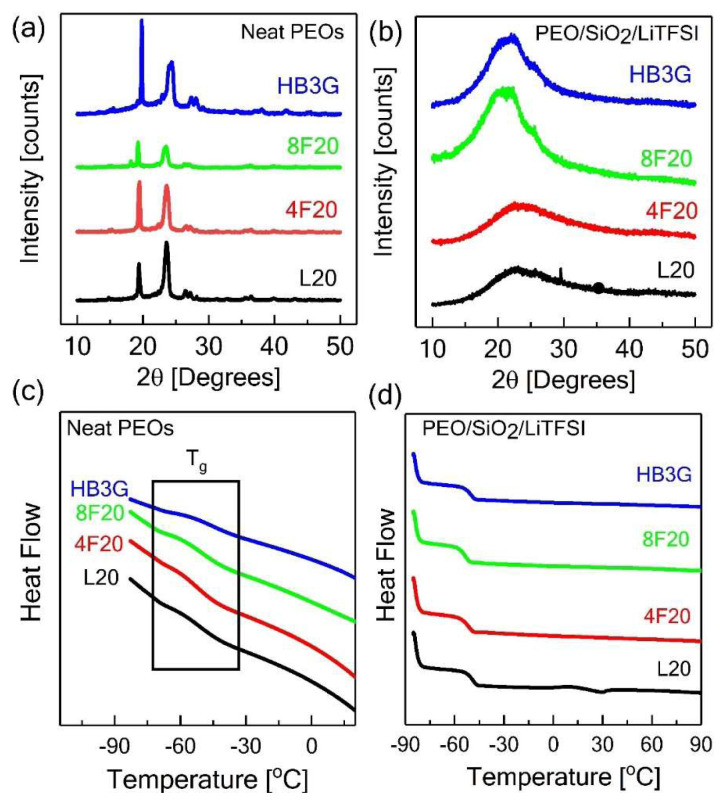
XRD and DSC results for neat PEOs (a), (c) and its respective PNCs based electrolytes (b), (d), respectively, containing 30 vol% silica nanoparticles and LiTFSI (Li/EO=0.085 MR) with various PEO topologies.

Next, the crystallization of the neat PEO homopolymers and polymer nanocomposite electrolytes involving 30 vol.% silica nanoparticles and LiTFSI ([Li/EO = 0.085]) were examined. Figures 3a and 3b show the room temperature X-ray diffraction (XRD) spectra for the neat PEOs with various topologies without salt and their corresponding PNC electrolytes. The intensity profiles of the pure PEOs with different architectures (Figure 3a) displayed primary peaks positioned at the same Bragg’s angles such as 2θ = 19° (120-plane) and 2θ = 23° (032-plane) due to the strong tendency of PEOs to crystallize at room temperature. However, crystal structure and interplanar distance remain identical for different PEO architectures [13, 33, 38, 39, 47]. Additionally, the degree of neat PEO crystallinity (% X_c_ = ΔH_m_/ΔH_c_ × 100) for different polymer topologies was estimated from the DSC thermograms given in Figure S2 by taking the ratio of the melting enthalpy (ΔH_m_) of the sample to the enthalpy of melting of completely crystalline PEO (ΔH_c_) which is taken as 196.4 J/g [13] (see Figure S2 in the Supporting Information for the DSC thermographs for neat PEOs with different architectures). In this context, when compared to linear counterpart, the melting enthalpies for the branched topologies considerably reduced, suggesting effective suppression of the PEO crystallization in these architectures because of the restricted mobility of the polymer chains to form crystal structures mainly due to the topological constraints introduced by the branching [39, 48, 49]. This reduction appears to be slightly dependent on the polymer architecture such that higher fraction of amorphous phase was formed with increased number of arms in these nonlinear architectures. This could be understood by the fact that lowering arm length arising from the increasing branching decreased the ability of the arms along with the slower crystallization kinetics in these nonlinear architectures in order to reorient for the formation of their own crystals [13, 39, 50]. Nevertheless, a significant level of crystal phase remains in all neat PEO architectures, which is not desirable for ionic conductivity.

As seen in Figure 3b, the incorporation of 30 vol% SiO_2_ nanoparticles and LiTFSI salt with a molar ratio (Li/EO = 0.085) resulted in a dramatic decrease in the peak intensity for L20/SiO_2_/LiTFSI composite electrolyte, whereas it completely prevented crystalline domains in the case of nonlinear PNCs-based electrolytes. The representative melting enthalpies and crystallinity ratios for neat PEOs with the architectural variations and their corresponding composite electrolytes are indicated by Table 2. Furthermore, the apparent melting temperature (T_m_) values estimated from DSC results for neat PEOs with various PEO topologies in Figure S2 were also architecture-dependent such that the nonlinear architectures had usually lower melting temperatures as the branching increased, which agrees with the previous findings [13, 42, 47]. The lower melting temperature of the branched architectures could be attributed to the thinning of the crystalline lamellae with slower crystallization rate as well as the increased ability for the salt dissolution arising from the stronger interaction between polymer chains and the lithium salt due to increased branching in these nonlinear topologies [13, 42, 47]. Overall, regardless of the topological variations, all these results suggest that the crystallinity of PEO can be remarkably inhibited in PEO-based electrolytes using well-dispersed SiO_2_ nanoparticles.

The effect of silica nanoparticles and salt on the glass transition temperature of the electrolytes was negligibly small. Figures 3c and 3d show the DSC thermograms in which the glass transition and melting process of both amorphous and crystalline phase is clearly noticed. As summarized in Table 1, the glass transition temperature of neat PEOs (T_g_ approximately −55 °C) (Figure 3c) showed no significance change with varying PEO architecture, implying preserved plasticity at room temperature. With the loadings of SiO_2_ NPs and LiTFSI salt (Figure 3d), we observed no significant variation in the glass transition temperature (T_g_) for the composite polymer electrolytes.

**Figure 4:**
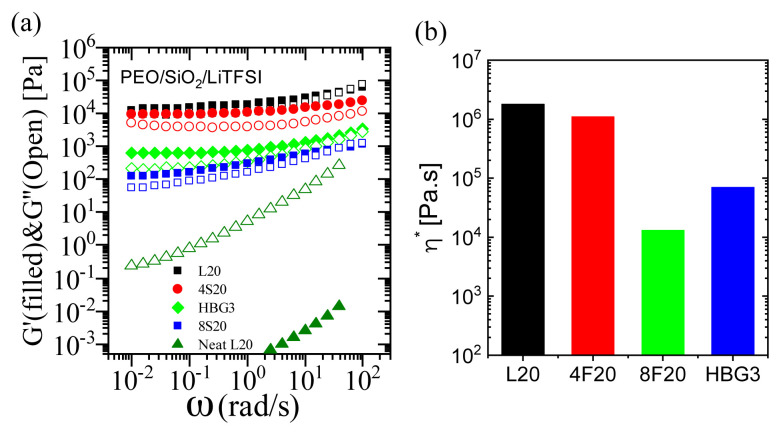
(a) Storage (G′) and loss (G″) moduli obtained from oscillatory shear measurements at 80 °C for the neat linear PEO and PEO-based composite electrolytes containing 30 vol % silica nanoparticles, and LiTFSI with MR = 0.085, (b) complex viscosity of the nanocomposite electrolytes.

Oscillatory shear rheology measurements on these composite polymer electrolytes helped to understand the relationships between their microscopic dynamics and the bulk transport properties. Linear viscoelastic moduli in small amplitude oscillatory shear regime for the composite electrolytes and neat linear polymer at 80 °C are presented in Figure 4a. Adding NPs to the polymer matrix significantly reinforces the PNC electrolytes and causes an increase in both storage (G′) and loss (G″) moduli. For all nanocomposite samples, G≥ exceeds G″, while making their frequency-dependence weaker, which is a characteristic of a gel-like solid behavior [51, 52]. The nanocomposite electrolyte with linear PEO shows the highest viscoelastic moduli compared to the branched architectures due to its flexible and entangling nature. 4-arms PEO matrix does not significantly change the moduli (although slightly lower values are noted), whereas the nanocomposite electrolytes with highly branched structures have significantly lower moduli. For example, the electrolyte with 8-arms star matrix has storage modulus that is two orders of magnitude lower compared to the nanocomposite sample with linear PEO. Figure 4b shows also the complex viscosity of the nanocomposite electrolytes. Overall, decreasing the arm length of the polymer chains lowers the interpenetrability of the chains which leads to lower entanglement and higher mobility. Among the PNC electrolytes with nonlinear matrices, 8-arms star has the most compact form with shortest arm length leading to lower interchain interactions in the matrix, hence induces the lowest viscoelastic moduli and viscosity. We emphasize that the sample is still highly reinforced and solid like as seen from more than three orders of magnitude shift in the low-frequency moduli compared to the neat PEO matrix. The nanocomposite electrolytes with branched architectures are therefore both solid-like and easier to process, which is desired for flexible batteries.

**Figure 5:**
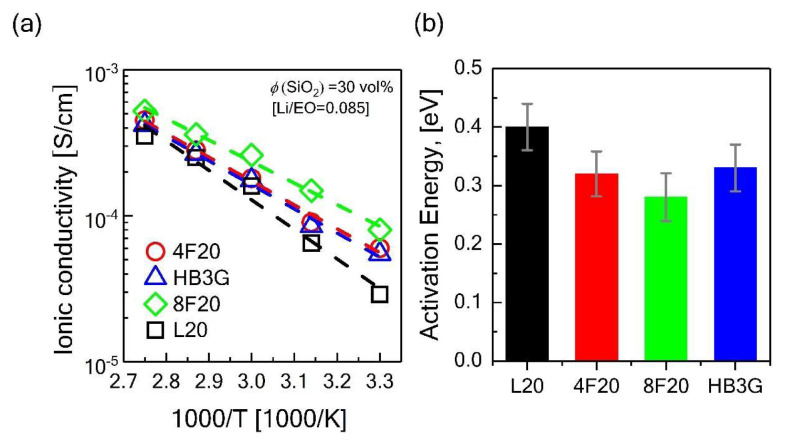
(a) Ionic conductivity measurements with corresponding Arrhenius fit lines as a function of varying temperatures between 30 and 90 °C with a temperature increase of 15 °C for different PEO architectures (b) estimated pseudo-activation energies for the PNCs-based electrolytes with various PEO architectures for the solid composite polymer electrolytes prepared using PEO, 30 vol% silica nanoparticles, and LiTFSI (0.085 MR).

To understand how the bulk rheological behavior reflects on the ionic conductivity, the electrochemical impedance spectroscopy (EIS) (an example of the Nyquist plots for the calculation details of the ionic conductivity are shown in Figure S3) experiments were performed. Figure 5 shows the ionic conductivities of the electrolytes at different temperatures between 30 and 90 °C with ΔT = 15 °C. Of all the electrolytes, the ones prepared with nonlinear topologies resulted in higher ionic conduction, which could be attributed to lower viscosity (Figure 4b) as well as higher free volume with the increased branching. More specifically, the enhancements in the ionic conductivity were approximately as high as 40% for 8-arm star, 28% for 4-arms star, and %16 for hyperbranched case in comparison with the composite with the linear matrix. Furthermore, the temperature dependence of ionic conductivity follows the Arrhenius behavior over the temperature range studied, indicating the ion transport is primarily controlled by the bulk diffusion in the viscous matrix, rather than ion-hopping [32]. This is primarily due to the fact that the time-scale of the segmental dynamics (which facilitates ion hopping between different coordination sites along the polymer backbone and between different chains) is too short as the samples are well-above their *T*_g_. The correlation between the bulk viscosity and the ion diffusion is also seen from the activation energies for the conduction. The Arrhenius fitting parameters for the ionic conductivity measurements with respect to temperature for the electrolytes are shown in Table S1. The Arrhenius model is given by [13],


(1) $$\sigma_{f}=\sigma_{0}\;\text{exp}\;E/RT$$

where σ_0_ is the reference conductivity, E is the active energy, and R is the gas constant number (8.314 J K^−^^1^ mol^−^^1^). Fitting the conductivity data to the Arrhenius model gives the estimated the pseudo-activation energies as shown in Figure 5b. The activation energies for ion hopping agrees well with the conductivity data, and lower activation barrier results in higher ionic conductivity [13]. The electrolyte with the linear PEO has an activation energy of 0.4 eV for the salt molar ratio of [Li/EO] = 0.085, which results in the lowest ionic conductivity among the PEO investigated architectures. On the other hand, activation energy decreased to 0.33, 0.32, and 0.29 eV for HB3G, 4-arms, and 8-arms star PEOs respectively which display higher ionic conductivity. This study shows that the activation energy for the conductivity in nanocomposite electrolytes is highly dependent on the architecture of the polymer used. Increasing branching enhances the polymer mobility in the nanocomposite electrolytes without sacrificing their solid-like nature, and thus allows obtaining higher ionic conductivity compared to the conventional nanocomposite electrolytes made of linear polymer chains.

## 4. Conclusion

In conclusion, new polymer nanocomposites (PNCs)-based electrolytes composed of a biocompatible and biodegradable polymer, PEO, with varying topologies (linear, stars, and hyperbranched), and silica nanoparticles with the addition of fixed amount LiTFSI were designed. It is clearly seen that PEO crystallization of the branched topologies with higher degree of branching was considerably reduced in comparison with the linear one, which facilitated the ionic conduction. Furthermore, the glass transition temperature (T_g_ approximately −55 °C) of nanocomposites showed no significant change with varying PEO architecture, suggesting preservation of the flexible nature at room temperature for nonlinear PEOs when compared to linear PEO. Furthermore, compared to the reported PEO-based polymer electrolytes in the literature, the addition of nanoparticles reinforced the electrolytes with more elasticity and on the other hand the idea of using nonlinear polymer chains as a host in the composite electrolytes significantly enhances the ion mobility in the matrix, when compared to the linear counterpart. In short, newly developed PEO-nanocomposite-based electrolytes provide significant advantages such as being more environmentally friendly, sustainable, and easily malleable. Consequently, obtained results for the composite electrolytes with branched PEOs show a new compelling direction for the applications especially including battery implants and wearable electronic which require flexibility, biocompatibility, and biodegradability.

## Supporting Information

Figure S1

Figure S2

Figure S3

## Figures and Tables

**Table 1:** Molecular characteristics including functionality, dispersity, total, and arm molecular weight of the PEO samples used in this study.

PEO architecture	Sample ID	Functionality (f)	MW [kg/mol]	M_arm_ [kg/mol]	Dispersity (*Đ*)
Linear	L20	2	20	10	1.10
4-arms star	4F20	4	20	5	1.03
8-arms star	8F20	8	20	2.5	1.10
Hyperbranched	HB3G	3rd generation	20	NA	<1.5

**Table 2:** Glass transition and melting temperatures with the estimated enthalpies and degree of crystallizations of various PEO architectures with 30 vol% Silica nanoparticles and constant lithium concentration (Li/EO = 0.085 MR).

Estimated parameters	PEO/silica nanoparticles/LiTFSI system				
	Li/EO	L20	4F20	8F20	HB3G
**T (melting temperature), °C**	0	57.8	55.3	48.4	51.2
	0.085	29	-	-	-
**T** **_g_** ** (glass transition), °C**	0	−51.1	−52.5	−51.0	−48.0
	0.085	−49.7	−51.7	−53.0	−51.0
**Melting enthalpy, J/g**	0	145.8	125.8	125.0	124.9
	0.085	1.1	-	-	-
**The degree of crystallization, %**	0	74.2	64.1	63.6	63.0
	0.085	0.6	-	-	-
