# Supplementary figures and images for: Enhanced ionic conductivity and mechanical strength in nanocomposite electrolytes with nonlinear polymer architectures
Source: Turk J Chem. 2022 Dec 12;47(1):242–52. doi: 10.55730/1300-0527.3533 (PMC10503998; doi:10.55730/1300-0527.3533)

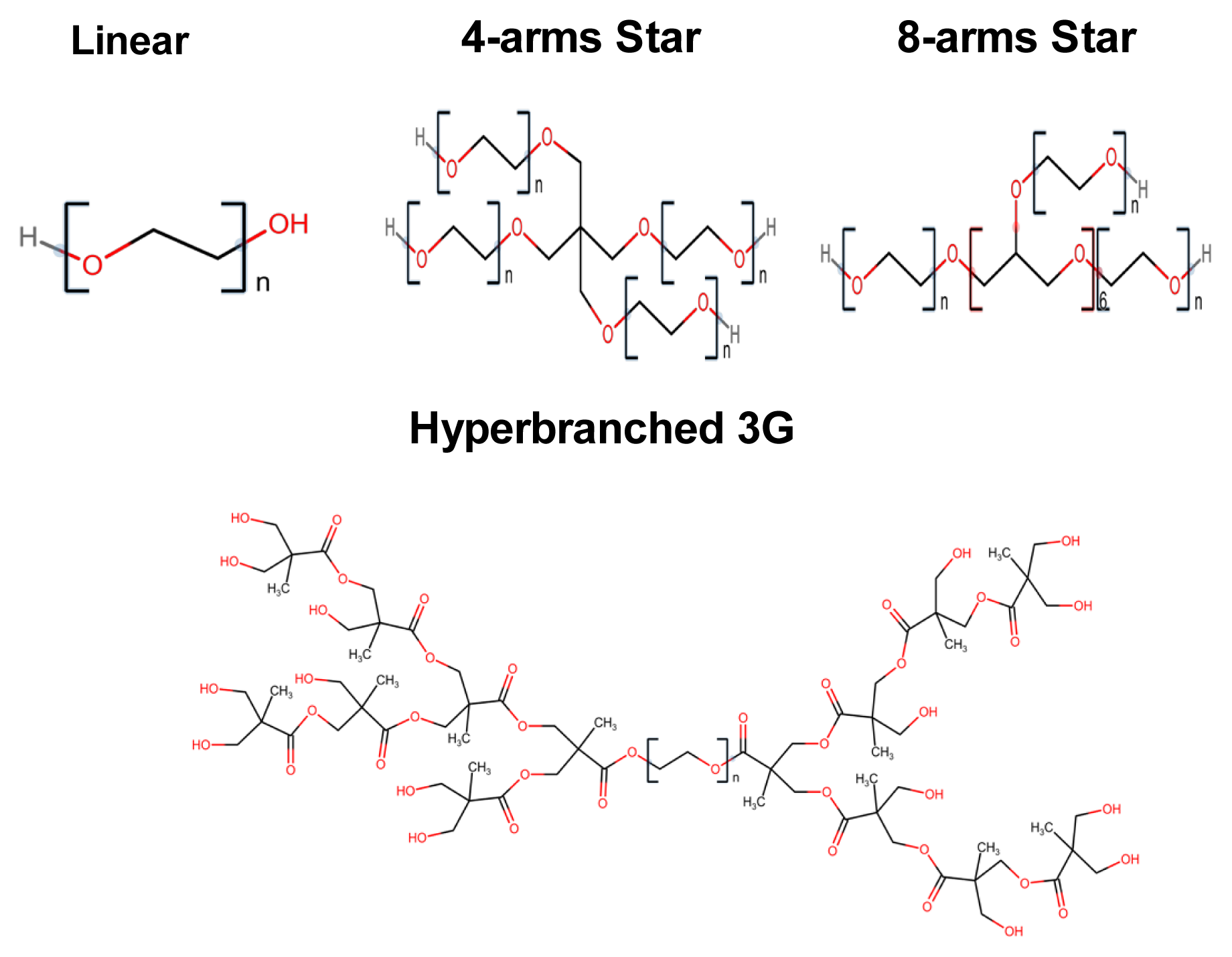

Supplement: Figure S1 [file turkjchem-47-1-242s1.tif]

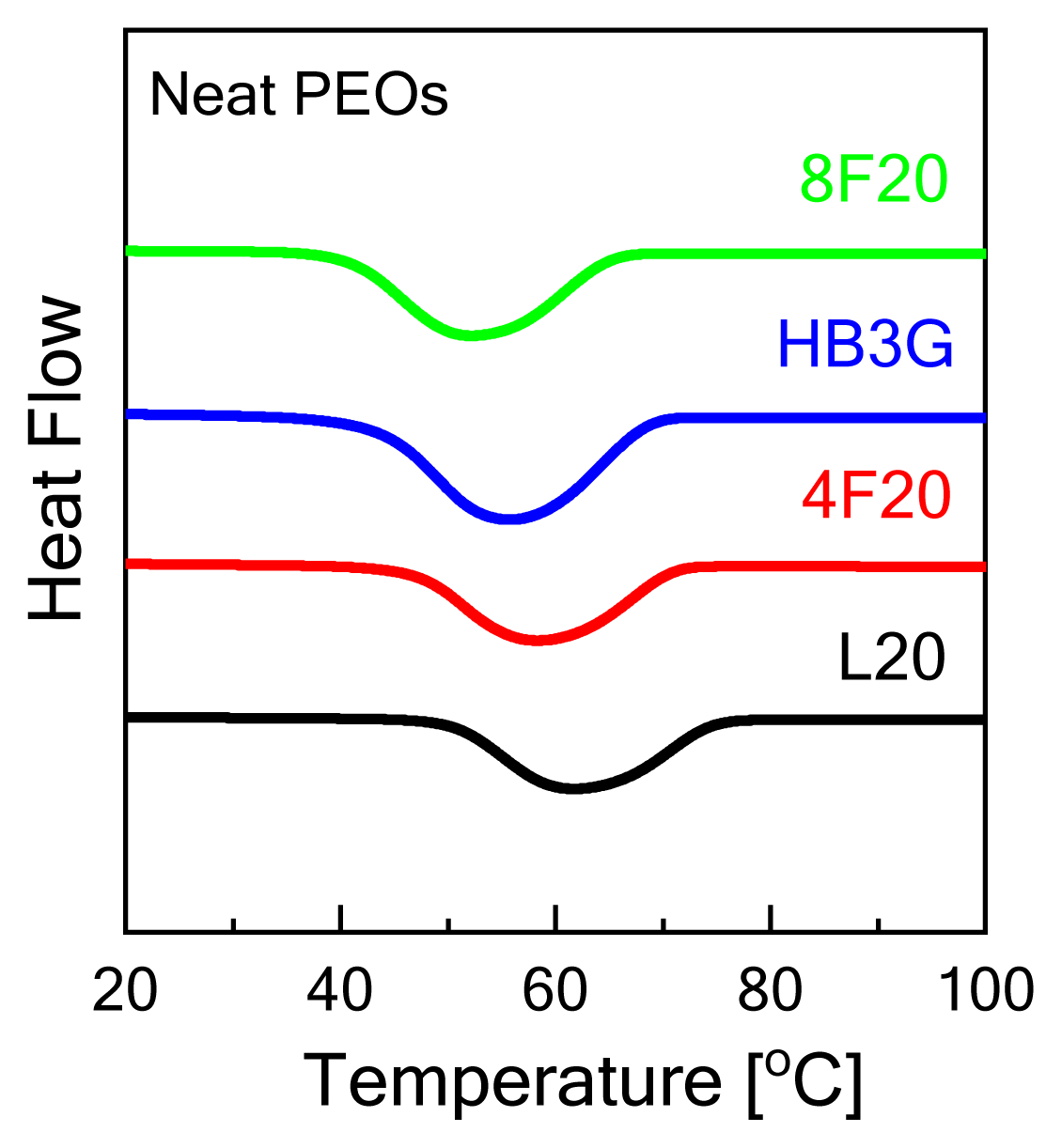

Supplement: Figure S2 [file turkjchem-47-1-242s2.tif]

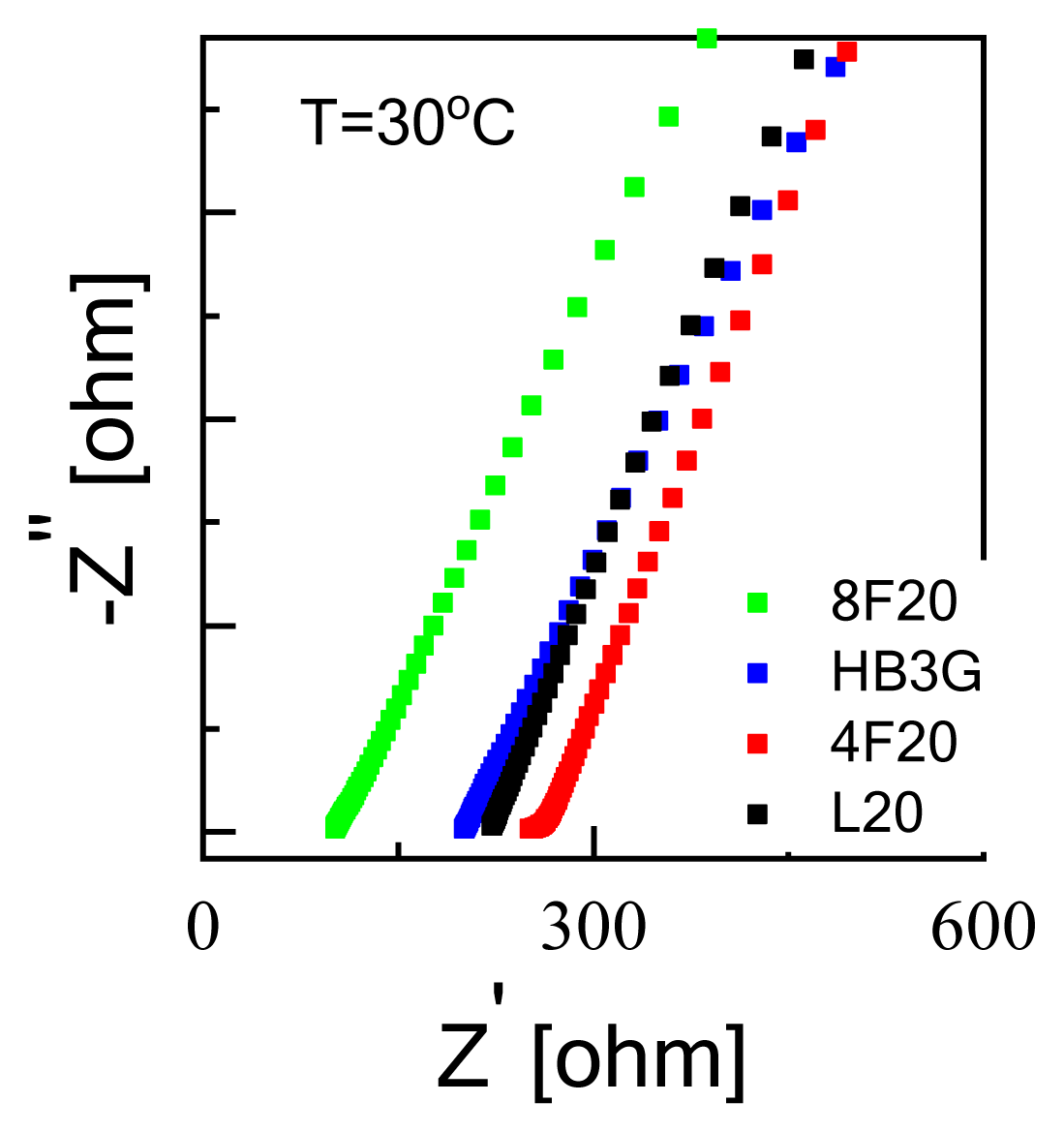

Supplement: Figure S3 [file turkjchem-47-1-242s3.tif]
